# Microbiota and Metabolomic Patterns in the Breast Milk of Subjects with Celiac Disease on a Gluten-Free Diet

**DOI:** 10.3390/nu13072243

**Published:** 2021-06-29

**Authors:** Katherine L. Olshan, Ali R. Zomorrodi, Meritxell Pujolassos, Jacopo Troisi, Nayeim Khan, Brian Fanelli, Victoria Kenyon, Alessio Fasano, Maureen M. Leonard

**Affiliations:** 1 Division of Pediatric Gastroenterology and Nutrition, MassGeneral Hospital for Children, Harvard Medical School, Boston, MA 02114, USA; kolshan@mgh.harvard.edu (K.L.O.); AZOMORRODI@mgh.harvard.edu (A.R.Z.); afasano@mgh.harvard.edu (A.F.); 2 Mucosal Immunology and Biology Research Center, MassGeneral Hospital for Children, Boston, MA 02129, USA; vakenyon@partners.org; 3 Department of Pediatrics, Harvard Medical School, Harvard University, Boston, MA 02115, USA; 4 Celiac Research Program, Harvard Medical School, Boston, MA 02115, USA; 5 Theoreo srl, University of Salerno, 84084 Salerno, Italy; meritxellpujolassos@gmail.com (M.P.); j.troisi@ebris.eu (J.T.); 6 Department of Medicine, Surgery and Dentistry, Scuola Medica Salernitana, University of Salerno, 84084 Salerno, Italy; 7 European Biomedical Research Institute of Salerno (EBRIS), Via S. De Renzi, 50, 84125 Salerno, Italy; 8 CosmosID Inc., Rockville, MD 20850, USA; nayeim@cosmosid.com (N.K.); brian.fanelli@cosmosid.com (B.F.)

**Keywords:** breast milk microbiome, celiac disease, multi-omics analysis

## Abstract

The intestinal microbiome may trigger celiac disease (CD) in individuals with a genetic disposition when exposed to dietary gluten. Research demonstrates that nutrition during infancy is crucial to the intestinal microbiome engraftment. Very few studies to date have focused on the breast milk composition of subjects with a history of CD on a gluten-free diet. Here, we utilize a multi-omics approach with shotgun metagenomics to analyze the breast milk microbiome integrated with metabolome profiling of 36 subjects, 20 with CD on a gluten-free diet and 16 healthy controls. These analyses identified significant differences in bacterial and viral species/strains and functional pathways but no difference in metabolite abundance. Specifically, three bacterial strains with increased abundance were identified in subjects with CD on a gluten-free diet of which one (*Rothia mucilaginosa*) has been previously linked to autoimmune conditions. We also identified five pathways with increased abundance in subjects with CD on a gluten-free diet. We additionally found four bacterial and two viral species/strains with increased abundance in healthy controls. Overall, the differences observed in bacterial and viral species/strains and in functional pathways observed in our analysis may influence microbiome engraftment in neonates, which may impact their future clinical outcomes.

## 1. Introduction

Celiac disease (CD) is an autoimmune enteropathy triggered by ingestion of gluten, a protein found in wheat, rye, and barley [1]. This disease occurs only in individuals with specific human leukocyte antigen (HLA) DQ haplotypes (DQ2, DQ8) [1]. However, while 30% of the population carries these compatible genetics, only 2–3% of these individuals ultimately develop CD [2]. This implies that genetic predisposition and exposure to dietary gluten are necessary but not sufficient to develop CD. Multiple environmental factors [3,4,5,6,7] including infant feeding type have been evaluated independently in case-control studies [3,4] and meta-analyses [8,9]. While early studies suggest that formula feeding during infancy is associated with an increased risk of developing CD [8], subsequent prospective studies have not found that breastfeeding was protective against developing CD [3,4].

Mounting evidence supports the role of the gut microbiota as one of the environmental factors involved in the pathogenesis of chronic medical conditions, including food allergy [5] and inflammatory bowel disease [6]. Many factors are known to alter the composition of the intestinal microbiota, including exposure to antibiotics [10], exposure to other medications [11,12], and dietary patterns [13]. Additionally, research suggests that there are significant differences in the intestinal microbiota of infants who are breastfed when compared with those who are formula fed, both in the general population [14,15] and in infants at risk for developing CD [16,17,18]. 

While human breast milk was once considered a sterile fluid, it is now recognized to contain a unique microbiota that likely plays a role in engraftment of the infant intestinal microbiota. The source of the breast milk microbiota is not fully understood, though is thought to be multifactorial and include the maternal gastrointestinal tract, the maternal skin, and the infant oropharynx [19]. Multiple studies have been conducted evaluating the microbial characteristics of breast milk in healthy subjects, and have identified the presence of bacterial species such as *Bifidobacterium* [20,21,22] (including *B. breve, B. adolescentis*, and *B. bifidum* [20]), *Lactobacillus* [22] (including, *L. rhamnosus* [23], *L. gasseri* [24,25], and *L. lactis* [23]), *Staphylococcus* [22], and *Streptococcus* [22]. However, few studies have been performed in mothers with CD. One case-control study utilized polymerase chain reaction (PCR)-based techniques and found a reduced abundance of *Bifidobacterium* species in mothers with CD when compared with mothers without CD at one month after delivery [26]. Another study compared the breast milk microbiota of subject’s whose children later did or did not develop CD at 9 months after delivery using 16S rRNA sequencing [27]. While they did not identify significant differences between the breast milk microbial composition in subjects with or without CD, they found that the breast milk of subjects whose children later developed CD had an increased abundance of *Methylobacterium komagatae, Methylocapsa palsarum*, and *Bacteroides vulgatus* [27]. 

Though these studies provide an important foundation for our understanding of the composition of the breast milk microbiota in CD, they do not focus on the initial period of breast feeding immediately following birth. Furthermore, previous studies utilize techniques such as PCR and 16S rRNA sequencing which are limited to identifying bacteria at the species level and do not allow for the identification of additional microorganisms (including viruses, protists, archaea, and fungi), nor can they directly provide information about the functional characterization of the microbiome. To our knowledge, metagenomic sequencing has not been utilized to investigate the breast milk microbiota of subjects with and without CD, which is of clinical interest given implications of the breast milk composition on the engraftment of the infant intestinal microbiota.

Here, we analyze the breast milk microbiota and metabolome of subjects with CD on a gluten-free diet and healthy controls as part of an ongoing prospective cohort study called the Celiac Disease Genomic, Environmental, Microbiome, and Metabolomic study (CDGEMM) [28], which follows over 500 infants at high risk of developing CD. We perform metagenomic and metabolomic analysis to compare samples collected one week after parturition in order to investigate whether there are differences in the breast milk of subjects with CD on a gluten-free diet compared with healthy control subjects who ingest gluten.

## 2. Materials and Methods

The CDGEMM cohort consists of 500 infants from the United States, Italy, and Spain with a first-degree relative with CD, who have been followed prospectively since birth [28]. As part of this study, we collect perinatal and maternal health information, as well as maternal breast milk and maternal pre-and post-natal stool, in addition to infant health and dietary information and infant samples (blood, stool). Thirty-six subjects from the United States were chosen for our analysis; 20 subjects with CD on a gluten-free diet and 16 healthy control subjects who ingest gluten. Parents of the infants included in the study provided written informed consent per the standards outlined and approved by the Partners Human Research Committee Institutional Review Board. Parents completed a detailed questionnaire at enrollment, providing information about pregnancy, delivery, maternal medical history, maternal antibiotic and probiotic usage, and infant antibiotic usage. 

All subjects included in our analysis provided a breast milk sample at 7–14 days after parturition. Maternal breast milk samples were collected using a pump at home, poured into the provided tube (10 mL), and immediately frozen for shipment. After overnight shipment, samples were placed in the −80 °C freezer for long-term storage. At the time of analysis, breast milk samples were thawed on ice and aliquoted. 

Maternal breast milk DNA was isolated using a modified INSPIRE protocol [29]. Given the naturally low biomass of microbial DNA in breast milk and the need for focus on sterile and validated processing techniques, we chose this well-known protocol previously utilized [30,31]. Briefly, samples (2 mL) were centrifuged (13,000× *g*) for 10 min at 4 °C. The fat layer was removed using a sterile swab, and the supernatant was discarded. The cell pellet was then resuspended in 500 mL TE 50 Enzyme dilution buffer. For enzymatic lysis, each sample was mixed with 100 mL of lytic enzyme cocktail mix. Lytic enzyme cocktail mix was composed of 50 mL lysozyme (lysozyme, 10 mg/mL ~400–500 KU/mL in molecular grade water; cat# L6876-10G, Sigma-Aldrich, Milwaukee, WI, USA), 6 mL mutanolysin (mutanolysin, 25 KU/mL in molecular grade water; cat# M9901-50KU, Sigma-Aldrich, Milwaukee, WI, USA), 3 mL lysostaphin (lysostaphin, 4000 U/mL in 20 mM sodium acetate; cat# L9043-5MG, Sigma-Aldrich, Milwaukee, WI, USA), and 41 mL TE 50 Enzyme dilution buffer. After resuspension in the lytic enzyme cocktail mix, the lysate mixture was then incubated on a dry heat block at 37 °C for one hour. A modified protocol for the QIAamp DNA Mini Kit was then utilized for DNA extraction [29].

The CosmosID (CosmosID Inc., Rockville, MD, USA) commercial metagenomic analysis platform (formerly known as GENIUS https://app.cosmosid.com/, accessed on 5 May 2020) [32,33] was used to identify the composition of breast milk microbiota up to a strain-level resolution as detailed in our previous work (Supplementary File S4) [18]. Functional profiling of metagenomic reads was also conducted by using the same platform, which works as follows: initial quality control, adapter trimming, and preprocessing of metagenomic sequencing reads are done using BBduk. The quality-controlled reads are then subjected to a translated search using Diamond BLASTX against a comprehensive and non-redundant protein sequence database, UniRef90. The mapping of metagenomic reads to gene sequences are weighted by mapping quality, coverage, and gene sequence length to estimate community-wide weighted gene family abundances as described by Franzosa et al. [34]. Gene families are then annotated to MetaCyc reactions (Metabolic Enzymes) to reconstruct and quantify MetaCyc metabolic pathways in the community as described in [34]. Furthermore, the UniRef90 gene families are regrouped to GO terms in order to get an overview of GO functions in the community. Lastly, to facilitate comparisons across multiple samples with different sequencing depths, the abundance values are normalized using Total-Sum Scaling (TSS) normalization to produce “copies per million” (analogous to TPMs in RNA-Seq) units. 

All human breast milk samples for metabolomics were processed using the MetaboPrep GC kit (Theoreo, Montecorvino Pugliano, Italy) according to the manufacturer instructions for the metabolome extraction, purification, and derivatization in preparation for gas chromatography–mass spectrometry (GC-MS) analysis in accordance with previous work (Supplementary File S4) [18]. A max tolerance of 50 for the linear index was used in this study. 

Chao1 richness estimator and Shannon diversity index for alpha diversity analysis were calculated using *estimateR* and *diversity* functions of the *vegan* R package [35], respectively. Bray–Curtis beta diversity analysis was conducted using *vegdist* and *pco* functions of *ecodist* R package [36]. 

Mann–Whitney *U* (Wilcoxon rank-sum) test (using the *wilcox* function of R) was used to identify microbes, pathways, and metabolites whose abundance is significantly different between subjects with a history of CD on a gluten-free diet and healthy controls. Significant results were reported for a *p*-value of <0.05 without adjustment for multiple testing. The corresponding adjusted *p*-values based on the Benjamini–Hochberg method (using *p.adjust* function of R with ‘fdr’ for its *method* argument) for these results are provided in Supplementary File S3.

## 3. Results

Thirty-six subjects with transitional breast milk samples available at 7–14 days after parturition were selected for analysis. Of those selected, 20 subjects had CD and were on a gluten-free diet; the other 16 were healthy control subjects ingesting gluten. Detailed information about pregnancy/delivery, maternal medical history, and maternal antibiotic and probiotic use (both during pregnancy and after delivery while breastfeeding) was collected (Table 1; Supplementary File S2).

Taxonomic profiling of the metagenomes was performed at both species- and strain-level resolution for bacteria, fungi, protists, and viruses (Supplementary File S1 and Figure S3). Functional profiling was also done to identify functional (MetaCyc) pathways encoded by each metagenome, and metabolomics analysis was conducted to profile the metabolites present in each sample (Supplementary Figures S1 and S2). No significant change in the abundance of fungi, protists, and metabolites was found in our analysis. 

### 3.1. Bacterial Composition

**Figure 1:**
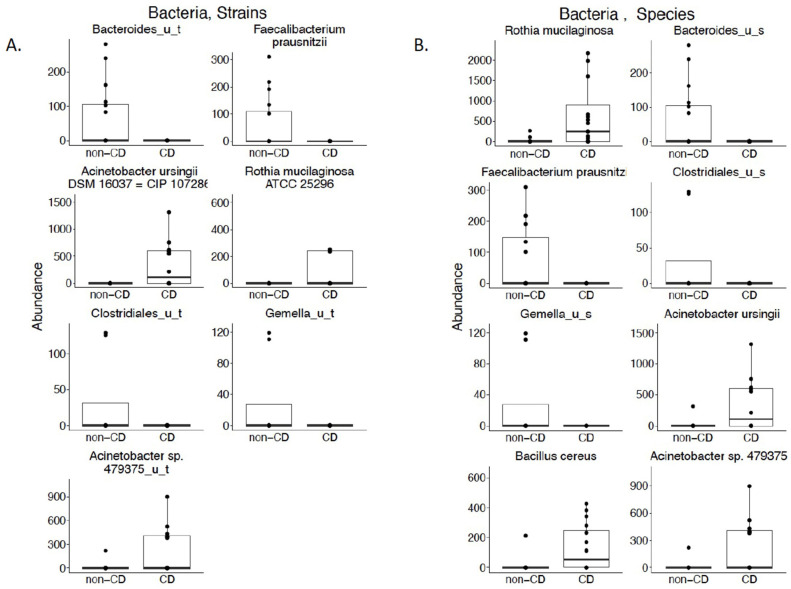
(**A**) Cross-sectional analysis of bacterial strains in the breast milk of subjects with CD on a gluten-free diet compared with healthy controls. Bacterial strains with a statistically significant difference in abundance between the breast milk of subjects with CD on a gluten-free diet and healthy controls according to Mann–Whitney *U* test (Wilcoxon rank-sum test) (*p*-value < 0.05); (**B**) Cross-sectional analysis of bacterial species in the breast milk of subjects with CD on a gluten-free diet compared with healthy controls. Bacterial species with a statistically significant difference in abundance between the breast milk of subjects with CD on a gluten-free diet and healthy controls according to Mann–Whitney *U* test (Wilcoxon rank-sum test) (*p*-value < 0.05).

We performed a cross-sectional analysis between subjects to evaluate differences in microbiota composition between our groups. We did not identify any differences in alpha or beta diversity at the species or strain level (*p*-value < 0.05; Supplementary Figures S1 and S2). We did, however, identify three bacterial strains with increased abundance in the breast milk of subjects with CD on a gluten-free diet: *Acinetobacter ursingii SM 16,037 = CIP 107286*, *Rothia mucilaginosa ATC 25296*, and *Acintobacter sp. 479375_u_t* (Figure 1A; *p*-value < 0.05). In addition to identifying an increased abundance in the corresponding species for these three strains, we also identified an increase in abundance of one other species, *Bacillus cereus* (Figure 1B; *p*-value < 0.05). We also identified four bacterial strains that were significantly increased in abundance in the breast milk of our healthy control subjects: *Bacteroides_u_t*, *Faecalibacterium prausnitzii*, *Clostridiales_u_t*, and *Gemella_u_t* (Figure 1A; *p*-value < 0.05). We also observed an increase in the abundance of species corresponding to these four strains (Figure 1B; *p*-value < 0.05). The “_u_t” and “_u_s” mean unspecified species and strains, respectively. This means that these taxa could not be resolved at the species or strain levels. 

### 3.2. Virome

**Figure 2:**
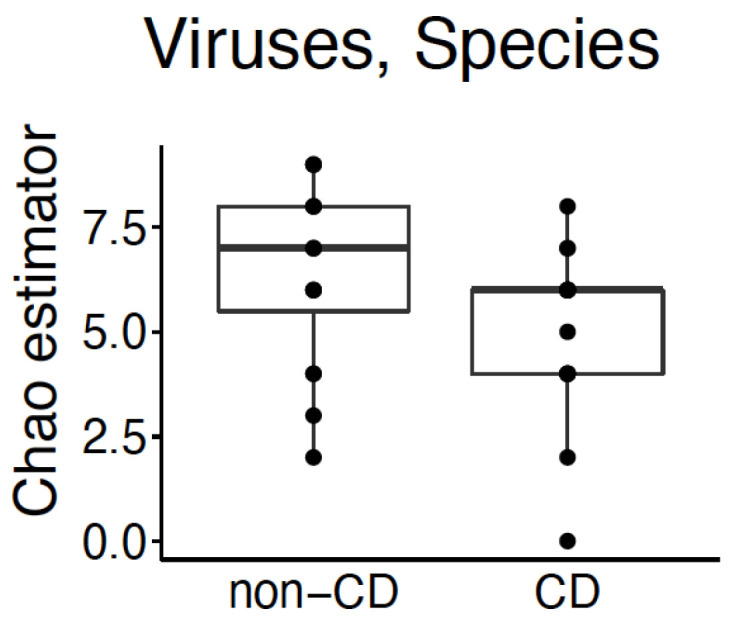
Cross-sectional analysis of viral species’ alpha diversity in the breast milk of subjects with CD on a gluten-free diet compared with healthy controls. Statistically significant difference in alpha diversity of viral species between the breast milk of subjects with CD on a gluten-free diet and healthy controls based on Chao1 estimator (*p*-value < 0.05).

**Figure 3:**
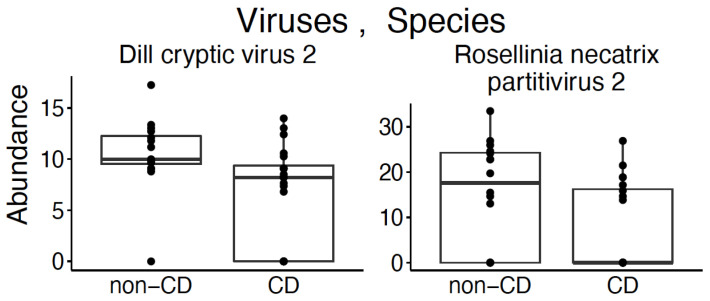
Cross-sectional analysis of viral species in the breast milk of subjects with CD on a gluten-free diet compared with healthy controls. Viral species with a statistically significant difference in abundance between the breast milk of subjects with CD on a gluten-free diet and healthy controls according to Mann–Whitney *U* test (Wilcoxon rank-sum test) (*p*-value < 0.05).

We identified a statistically significant difference in the alpha diversity between viral species (Figure 2; *p*-value < 0.05) using the Chao1 estimator to evaluate differences between groups. There was no identified difference in beta diversity (Supplementary Figure S2). Two viral species were found in increased abundance in healthy control subjects: *Dill cryptic virus 2* and *Rosellinia necatrix partitivirus 2* (Figure 3; *p*-value < 0.05). 

### 3.3. Pathways

**Figure 4:**
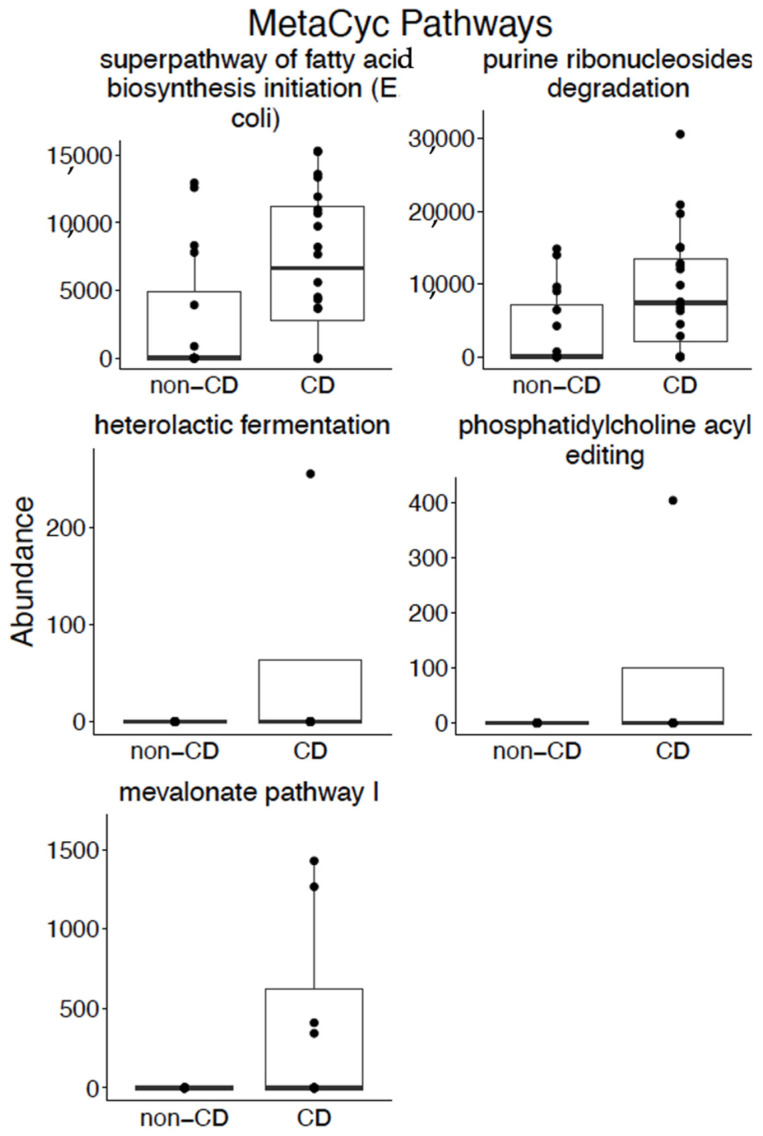
Cross-sectional analysis of MetaCyc pathways in the breast milk of subjects with CD on a gluten-free diet compared with healthy controls. Pathways with a statistically significant difference in abundance between the breast milk of subjects with CD on a gluten-free diet and healthy controls according to Mann–Whitney *U* test (Wilcoxon rank-sum test) (*p*-value < 0.05).

Our cross-sectional analysis identified five pathways with increased abundance in subjects with CD on a gluten-free diet. These pathways included: superpathway of fatty acid biosynthesis initiation (*E*. *coli*), purine ribonucleosides degradation, heterolactic fermentation, phosphatidylcholine acyl editing, and mevalonate pathway I (Figure 4; *p*-value < 0.05).

## 4. Discussion

To our knowledge, metagenomic sequencing has not been employed to explore the differences in the microbial and metabolomic composition of breast milk in subjects with CD. Our analysis provides novel insights into differences at the species and strain level for bacteria and viruses. We found that breast milk composition of subjects with CD on a gluten-free diet appears to be quite similar to the breast milk composition of healthy control subjects at 7–14 days post-partum. There was no difference in diversity for bacteria; however, we did identify a difference in alpha diversity for viruses. We also identified differences between the breast milk of subjects with CD on a gluten-free diet and healthy controls at both the strain and the species level for bacteria and viruses. 

Given that the majority of available microbiome and virome literature at this time is at the species level, here we focus the discussion of our results at the species level. While a previous study which utilized 16S sequencing did not identify differences in the breast milk microbiota of subjects with CD compared with healthy controls [27], we identified statistically significant differences in eight bacterial species and two viral species between the two groups. The eight bacteria isolated in our study have previously been isolated from the breast milk of healthy subjects [37,38,39,40,41,42,43,44]. We did not identify breast milk literature for these bacteria in CD or other treated or untreated diseases; however, we did identify literature related to the intestinal microbiota for some of these bacteria. For example, *Rothia mucilaginosa*, which was noted to have an increased abundance in the breast milk of subjects with CD, is also reported to have an increased abundance in the gut microbiota of subjects with autoimmune inflammatory conditions such as primary sclerosing cholangitis [45]. Additionally, we identified an increased abundance of *Faecalibacterium prausnitzii* in the breast milk of healthy control subjects, which has been found to be in decreased abundance in the intestinal microbiome of subjects with active inflammatory bowel disease [46]. Our virome analysis found two species, *Dill cryptic virus 2* and *Rosellinia necatrix partitivirus 2*, increased in abundance in the breast milk of healthy control subjects. To our knowledge, there is no prior published data of the breast milk virome in disease processes for comparison. We did identify five pathways with increased abundance in subjects with CD on a gluten-free diet. Heterolactic fermentation was previously linked to gastrointestinal disease with an increased abundance in esophageal brushings of patients with Barrett’s esophagus when compared with healthy controls [47]. Otherwise, there is no prior research reporting these pathways, and they may be CD specific, though more research is required to further elucidate these relationships. 

While we were able to identify statistically significant differences between subjects with CD on a gluten-free diet and healthy control subjects, our study is limited by a relatively small sample size. This implies that a number of differentially abundant features reported in this study based on unadjusted *p*-values for multiple testing have a high chance of being false positives (see Supplementary File S3 for a list of features with a high false discovery rate). This small sample size was chosen for our pilot analysis and can be expanded upon in the future due to ongoing enrollment in this study. Furthermore, as our subjects with CD were on a gluten-free diet and possibly in remission, it would be valuable also to compare the results of our breast milk microbiome and metabolome analyses with individuals with active CD or healthy individuals on a gluten-free diet for reasons other than CD, to see whether additional or distinct differences might be identified. These comparisons would provide insight into whether the differences noted in our analysis were secondary to disease state (CD on a gluten-free diet and possibly in remission) or diet (gluten-free). Finally, our study is a case-control model, which allows for an initial snapshot of the state of the breast milk microenvironment at a single point in time. It will be important in future prospective studies to consider longitudinal data to identify trends throughout the stages of breastfeeding. 

## 5. Conclusions

In this manuscript, we utilized a comprehensive metagenomic approach to evaluate the composition of transitional breast milk in subjects with and without CD, and we identified multiple bacterial strains, species, and viral species that differ between these two groups. To the best of our knowledge, there is little other literature evidence demonstrating similar trends, thus supporting the need for further breast milk and intestinal microbiome research, both in CD and in other disease processes. Additionally, further work is required to investigate whether the differences we noted impact the intestinal engraftment in the offspring, and thus potentially impact the long-term health of the offspring.

## Figures and Tables

**Table 1:** Study cohort metadata.

	Healthy Controls (*n* = 16)	Subjects with CD on a Gluten-Free Diet (*n* = 20)
Maternal Characteristics		
Age (years) *	33.4	32.5
Duration gestation (weeks) *	39.9	39.4
Pregnancy (%)		
Nulliparous	3 (18.8)	6 (30)
Multiparous	13 (81.3)	14 (70)
Mode of delivery (%)		
Vaginal	13 (81.3)	15 (75)
Cesarean	3 (18.8)	5 (25)
Antibiotics during pregnancy (%)	5 (31.3)	5 (25)
Antibiotics during delivery (%)	3 (18.8)	6 (30)
Probiotics during pregnancy (%)	7 (43.8)	5 (25)
Probiotics while breastfeeding (%)	5 (31.3)	4 (20)
Delivery setting (%)		
Hospital	15 (93.8)	19 (95)
Other (birthing center, home)	1 (6.2)	1 (5)
Pre-pregnancy body mass index (BMI) (kg/m^2^) *	22.4	23
Infant Characteristics		
Sex (%)		
Male	10 (62.5)	13 (65)
Female	6 (37.5)	7 (35)
Antibiotics at birth (%)	0 (0)	1 (5)
Duration of hospitalization (days) *	2.03	1.95

* Indicates average value.

## Data Availability

Publicly available datasets were analyzed in this study. This data can be found here: SRA SUB9917188.

## Supplementary Materials

The following are available online at https://www.mdpi.com/article/10.3390/nu13072243/s1, File S1: Results of the taxonomic, functional, and metabolomic profiling of breast milk samples; File S2: Subject characteristics; File S3: Unadjusted *p*-values of significant features (Wilcoxon, *p*-value < 0.5) for bacterial (strains, species), viral (strains, species), and pathways, and the corresponding *q*-values; File S4: Expanded Metagenomic and Metabolomic Methods; Figure S1: Alpha diversities (bacteria, fungi, protists, viruses); Figure S2: Beta diversities (bacteria, viruses); Figure S3: Taxonomic composition (bacteria, viruses, fungi, protists).

## Abbreviations

CD	celiac disease
HLA	human leukocyte antigen
PCR	polymerase chain reaction
GC-MS	gas chromatography–mass spectrometry
BMI	body mass index
