# Supplementary material for: Microbiota and Metabolomic Patterns in the Breast Milk of Subjects with Celiac Disease on a Gluten-Free Diet
Source: Nutrients. 2021 Jun 29;13(7):2243. doi: 10.3390/nu13072243 (PMC8308312; doi:10.3390/nu13072243)
Supplement: Supplementary file 1 [file nutrients-13-02243-s001.zip › supplementary/Supplementary Figure 3 - Stacked Bar Plots.pdf]

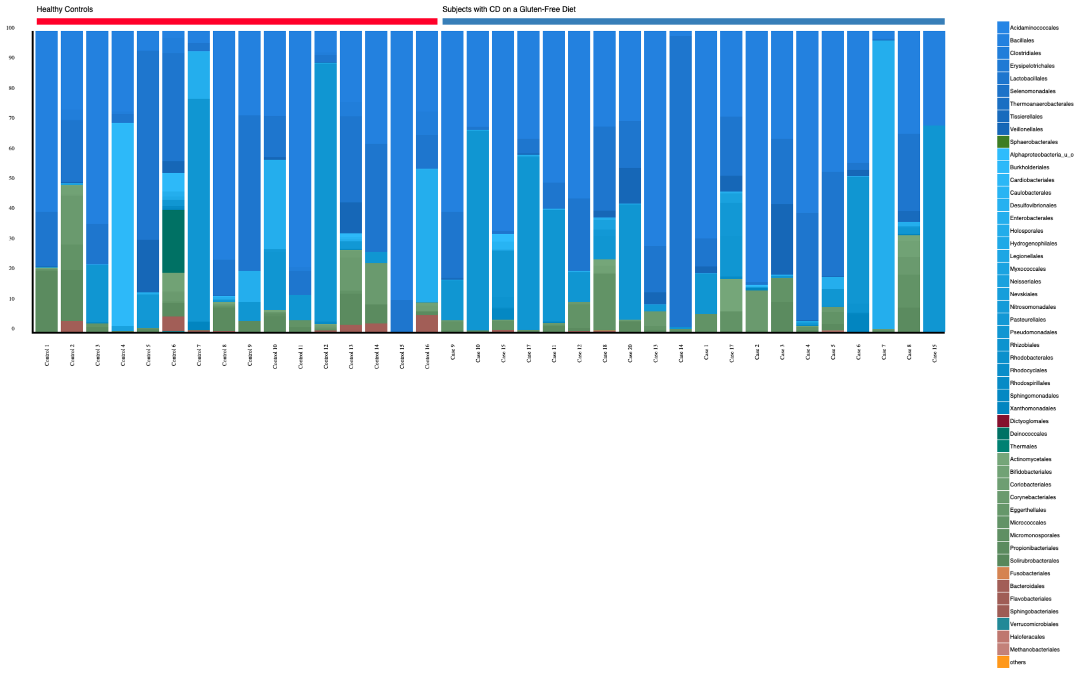
Healthy Controls
Subjects with CD on a Gluten-Free Diet
100
90
80
70
60
50
40
30
20
10
0
Control 1
Control 2
Control 3
Control 4
Control 5
Control 6
Control 7
Control 8
Control 9
Control 10
Control 11
Control 12
Control 13
Control 14
Control 15
Control 16
Case 9
Case 10
Case 15
Case 17
Case 11
Case 12
Case 18
Case 20
Case 13
Case 14
Case 1
Case 17
Case 2
Case 3
Case 4
Case 5
Case 6
Case 7
Case 8
Case 15
Acidaminococcales
Bacillales
Clostridiales
Erysipelotrichales
Lactobacillales
Selenomonadales
Thermoanaerobacterales
Tissierellales
Veillonellales
Sphaerobacterales
Alphaproteobacteria_u_o
Burkholderiales
Cardiobacteriales
Caulobacterales
Desulfovibrionales
Enterobacterales
Holosporales
Hydrogenophilales
Legionellales
Myxococcales
Neisseriales
Nevskiales
Nitrosomonadales
Pasteurellales
Pseudomonadales
Rhizobiales
Rhodobacterales
Rhodocyclales
Rhodospirillales
Sphingomonadales
Xanthomonadales
Dictyoglomales
Deinococcales
Thermales
Actinomycetales
Bifidobacteriales
Coriobacteriales
Corynebacteriales
Eggerthellales
Micrococcales
Micromonosporales
Propionibacteriales
Solirubrobacterales
Fusobacteriales
Bacteroidales
Flavobacteriales
Sphingobacteriales
Verrucomicrobiales
Haloferacales
Methanobacteriales
others

A.

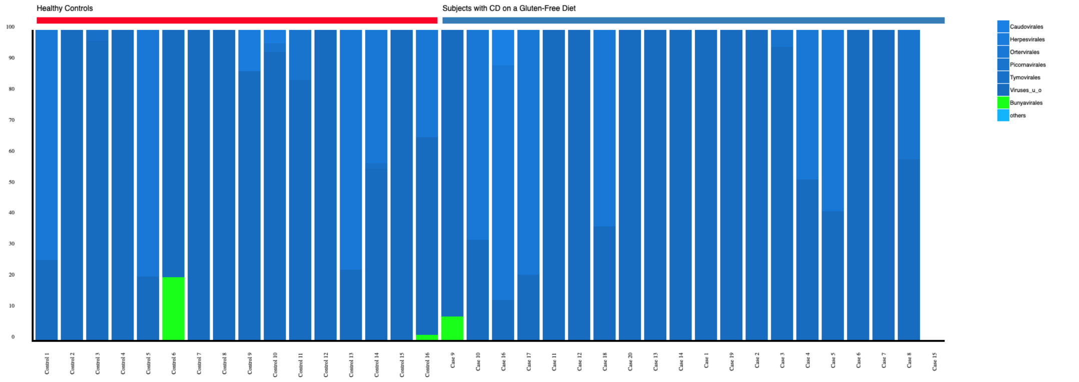
Healthy Controls
Subjects with CD on a Gluten-Free Diet
100
90
80
70
60
50
40
30
20
10
0
Control 1
Control 2
Control 3
Control 4
Control 5
Control 6
Control 7
Control 8
Control 9
Control 10
Control 11
Control 12
Control 13
Control 14
Control 15
Control 16
Case 9
Case 10
Case 16
Case 17
Case 11
Case 12
Case 18
Case 20
Case 13
Case 14
Case 1
Case 19
Case 2
Case 3
Case 4
Case 5
Case 6
Case 7
Case 8
Case 15
Caudovirales
Herpesvirales
Ortervirales
Picornavirales
Tymovirales
Viruses_u_o
Bunyavirales
others

B.

C.

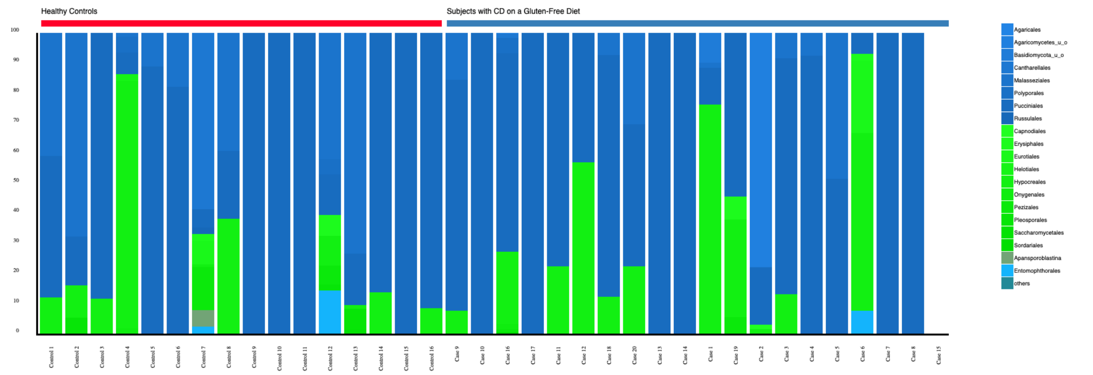

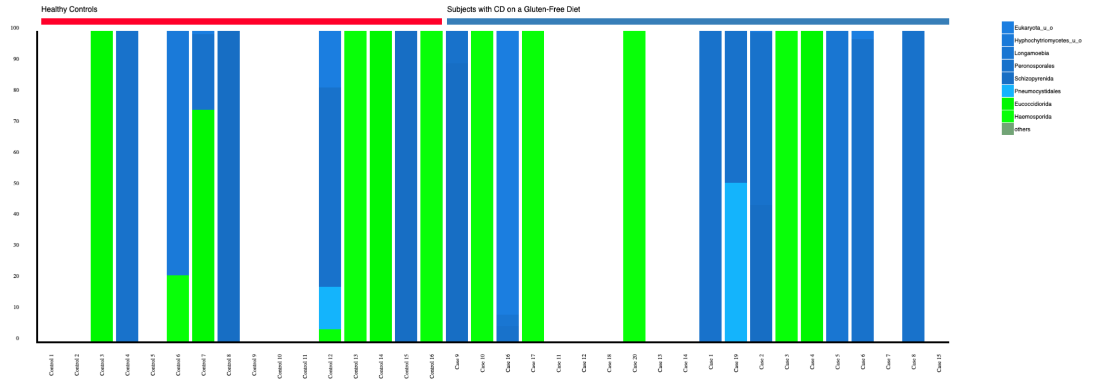

D.

Supplementary Figure S3: Taxonomic composition 36 breast milk samples at the order level for (A) bacteria, (B) viruses, (C) fungi and (D) protists.
