# Supplementary material for: Microbiota and Metabolomic Patterns in the Breast Milk of Subjects with Celiac Disease on a Gluten-Free Diet
Source: Nutrients. 2021 Jun 29;13(7):2243. doi: 10.3390/nu13072243 (PMC8308312; doi:10.3390/nu13072243)
Supplement: Supplementary file 1 [file nutrients-13-02243-s001.zip › supplementary/Supplementary File 4- Metagenomic and Metabolomic Sequencing Methods - Metagenomic and Metabolomic Methods.pdf]

Supplementary File 4: Metagenomic and Metabolomic Sequencing Methods [1]

“”Taxonomic profiling

Metagenomic sequencing reads were analyzed by using the CosmosID’s (CosmosID Inc., Rockville, MD) commercial metagenomic analysis platform (formerly knowns as GENIUS; https://app.cosmosid.com/login) [2,3] to reveal the underlying microbial community composition up to the species-level resolution (see Additional file 5 for a description of this platform).

Functional profiling

After trimming the raw sequencing reads using BBDuk (https://jgi.doe.gov/data-and-tools/bbtools/), we used the SPAdes tool [4] for the assembly of metagenomes and subsequently Prodigal [5] to identify protein coding sequences in the assembled metagenomes. We then utilized InterProScan [6] to annotate the identified genes with biochemical functions based on the KEGG pathways [7]. The relative abundance of each gene was computed as $G = \frac{L*C}{(R-K+1)}$, where, G is fragments per kilobase per million (FPKM) for each gene, L is the length of the gene, C is the coverage of contig in which the gene is identified, R is the read length and K is the k-mer size [8]. The relative abundance of each KEGG pathway was then quantified by summing the relative abundances of all the genes associated to that pathway.

Metabolomic profiling

All stool samples for metabolomics were stored and processed in Italy. The metabolome extraction, purification and derivatization were carried by the MetaboPrep GC kit (Theoreo, Montecorvino Pugliano, Italy) according to manufacturer instructions. Instrumental analyses were performed with a GC-MS system (GC-2010 Plus gas chromatograph and QP2010 Plus mass spectrometer; Shimadzu Corp., Kyoto, Japan). Sample analysis was performed in triplicate.

The molecular identity of metabolites was determined by analysis of the corresponding mass spectrum in the chromatogram, setting the linear index difference max tolerance to 10. These identified metabolites were further confirmed using external standards according to level 1 Metabolomics Standards Initiative (MSI) [9].””

References

1. Leonard, M.K., H; Pujolassos, M; Troisi, J; Valitutti, F; Subramanian, P; Camhi, S; Kenyon, V; Colucci, A; Serena, G; Cucchiara, S; Montuori, M; Malamisura, B; Francavilla, R; Elli, L; Fanelli, B; Colwell, R; Hasan, N; Zomorrodi, AR; Fasano, A; Team, Cdgemm Study. Multi-omics analysis reveals the influence of genetic and environmental risk factors on developing gut microbiota in infants at risk of celiac disease. *Microbiome* **2020**, doi:10.21203/rs.2.24237/v1.
2. Hasan, N.A.; Young, B.A.; Minard-Smith, A.T.; Saeed, K.; Li, H.; Heizer, E.M.; McMillan, N.J.; Isom, R.; Abdullah, A.S.; Bornman, D.M.; et al. Microbial community profiling of human saliva using shotgun metagenomic sequencing. *PLoS One* **2014**, *9*, e97699, doi:10.1371/journal.pone.0097699.
3. Ponnusamy, D.; Kozlova, E.V.; Sha, J.; Erova, T.E.; Azar, S.R.; Fitts, E.C.; Kirtley, M.L.; Tiner, B.L.; Andersson, J.A.; Grim, C.J.; et al. Cross-talk among flesh-eating Aeromonas hydrophila strains in mixed infection leading to necrotizing fasciitis. *Proc Natl Acad Sci U S A* **2016**, *113*, 722-727, doi:10.1073/pnas.1523817113.
4. Bankevich, A.; Nurk, S.; Antipov, D.; Gurevich, A.A.; Dvorkin, M.; Kulikov, A.S.; Lesin, V.M.; Nikolenko, S.I.; Pham, S.; Prjibelski, A.D. SPAdes: a new genome assembly algorithm and its applications to single-cell sequencing. *Journal of computational biology* **2012**, *19*, 455-477.
5. Hyatt, D.; Chen, G.-L.; LoCascio, P.F.; Land, M.L.; Larimer, F.W.; Hauser, L.J. Prodigal: prokaryotic gene recognition and translation initiation site identification. *BMC bioinformatics* **2010**, *11*, 119.
6. Jones, P.; Binns, D.; Chang, H.-Y.; Fraser, M.; Li, W.; McAnulla, C.; McWilliam, H.; Maslen, J.; Mitchell, A.; Nuka, G. InterProScan 5: genome-scale protein function classification. *Bioinformatics* **2014**, *30*, 1236-1240.
7. Kanehisa, M.; Goto, S. KEGG: kyoto encyclopedia of genes and genomes. *Nucleic acids research* **2000**, *28*, 27-30.
8. Zerbino, D.R.; Birney, E. Velvet: Algorithms for de novo short read assembly using de Bruijn. *Genome Research* **2004**.
9. Sumner, L.W.; Amberg, A.; Barrett, D.; Beale, M.H.; Beger, R.; Daykin, C.A.; Fan, T.W.-M.; Fiehn, O.; Goodacre, R.; Griffin, J.L. Proposed minimum reporting standards for chemical analysis. *Metabolomics* **2007**, *3*, 211-221.
