# Supplementary figures and images for: Microbiota and Metabolomic Patterns in the Breast Milk of Subjects with Celiac Disease on a Gluten-Free Diet
Source: Nutrients. 2021 Jun 29;13(7):2243. doi: 10.3390/nu13072243 (PMC8308312; doi:10.3390/nu13072243)

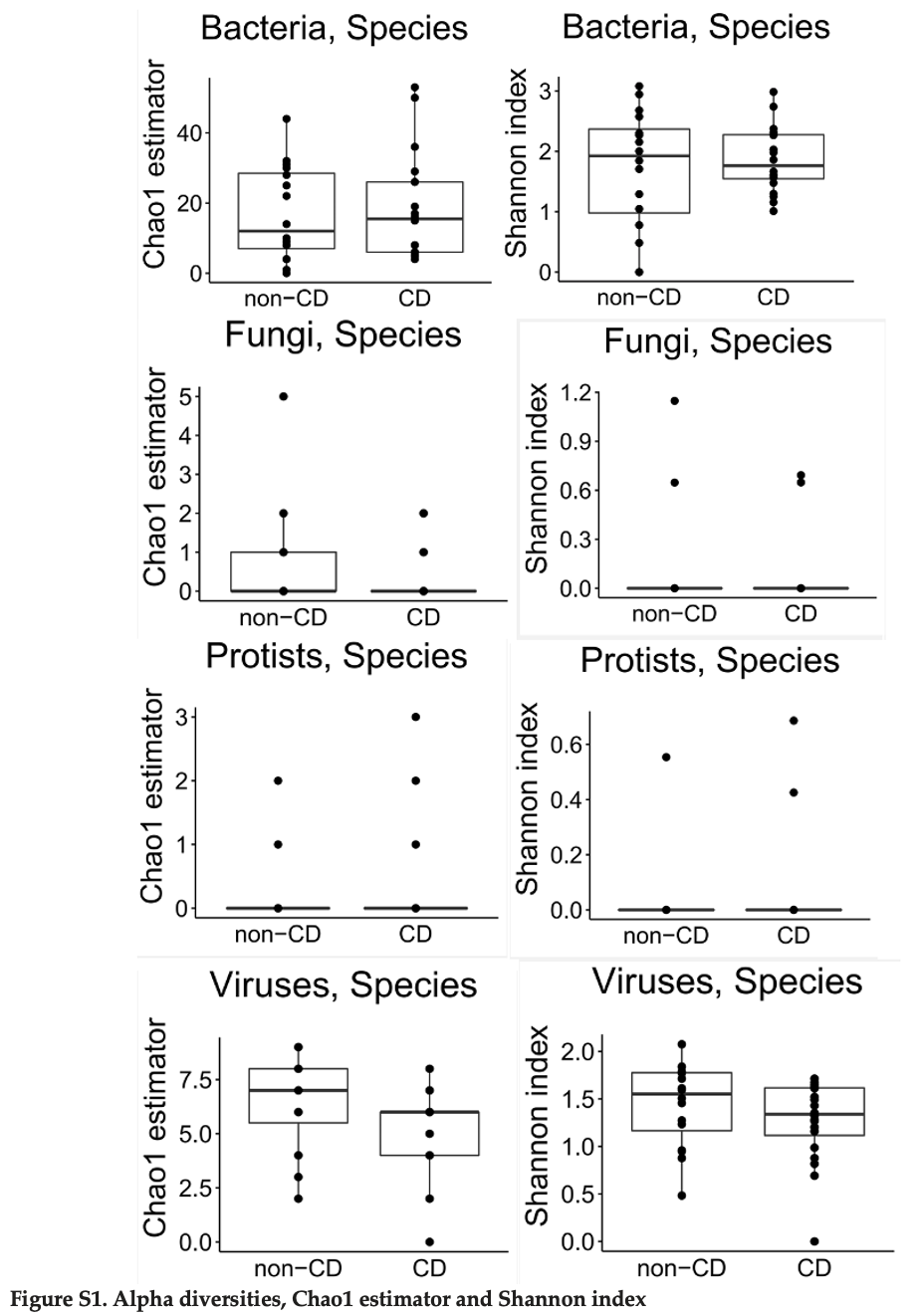

Supplement: Supplementary file 1 [file nutrients-13-02243-s001.zip › supplementary/Supplementary Figure 1 alpha diversities.png]

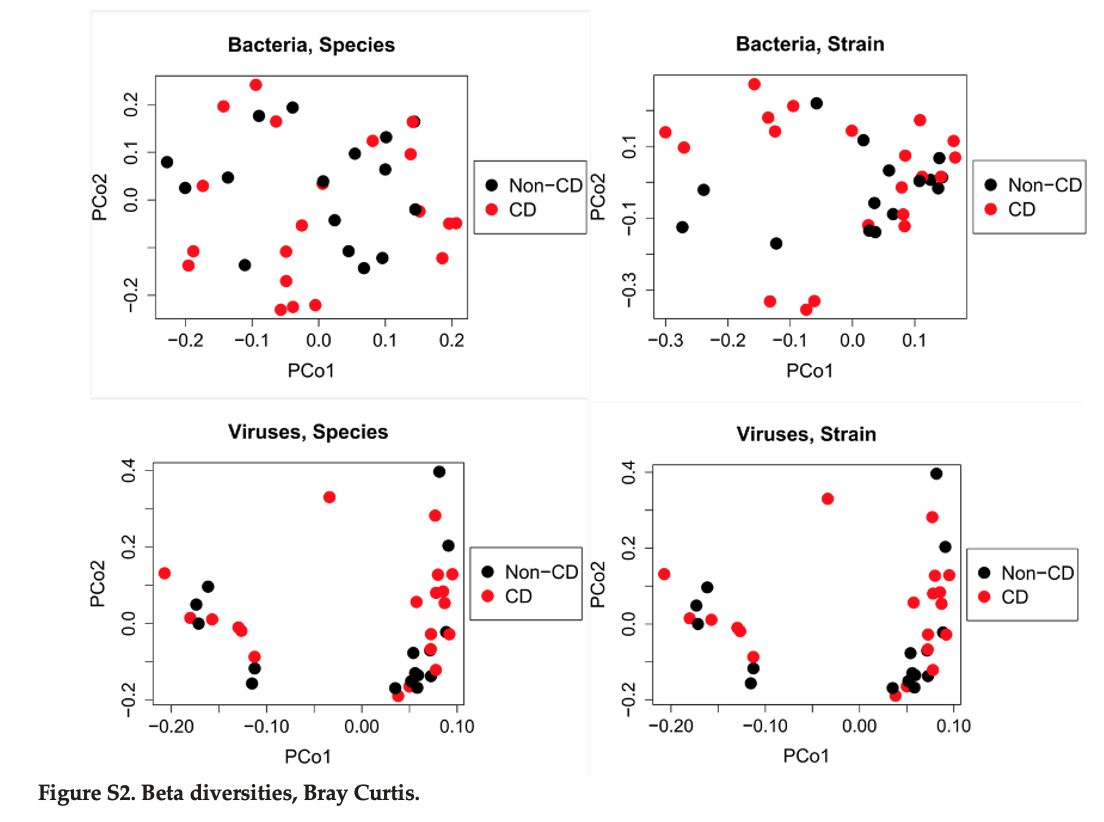

Supplement: Supplementary file 1 [file nutrients-13-02243-s001.zip › supplementary/Supplementary Figure 2 beta diversities.png]
